# The Sociodemographic Determinants of Health Literacy in the Ethnic Hungarian Mothers of Young Children in Eastern Europe

**DOI:** 10.3390/ijerph18115517

**Published:** 2021-05-21

**Authors:** Ágnes Sántha

**Affiliations:** Department of Applied Social Sciences, Faculty of Technical and Human Sciences, Sapientia Hungarian University of Transylvania, 540485 Tirgu Mures-Corunca 1C, Romania; santhaagnes@ms.sapientia.ro

**Keywords:** health literacy, BHLS, HLS-EU-16, social inequalities, health determinants, single mothers, young mothers

## Abstract

Parental health literacy is a decisive factor for child health and quality of life. Children of parents with limited health literacy are at increased risk of illness and longer recovery periods. The research at the Quality of Life Research Centre is aimed at studying the health literacy of ethnic Hungarian mothers in Eastern Europe (Hungary, Slovakia, Romania) as well as at assessing its socioeconomic and demographic antecedents. The sample size is 894 mothers. Our standardized online questionnaire includes the HLS-EU-16 and the BHLS questions, with the latter intended to screen for inadequate health literacy. Predictors of health literacy in mothers are socioeconomic status, age and partnership status. A key finding is the improvement of health literacy with age. Assessing the association of partnership status and health literacy is a novelty in this region. Our analysis reinforces the role of socioeconomic capital, widely recognized to be associated with health literacy in general and with parental health literacy in particular. Results indicate the necessity of improving caregiver health literacy with a range of health promotional activities in Eastern Europe, especially among mothers with low socioeconomic status. The hardships of young mothers and single mothers should also be considered in this respect.

## 1. Introduction

Health literacy has become widely acknowledged as a mediator between social status and health outcomes. Limited health literacy not only influences peoples’ behaviours related to self-management of chronic conditions, but also affects health outcomes [1]. This also applies to parents’ health literacy with respect to child health issues [2]. Child health indicators in Eastern Europe are unfavorable compared to the rest of the continent [3], which justifies the need to explore caregivers’ health literacy.

Eastern Europe was underrepresented and the Hungarian population not included in the European Health Literacy Survey of 2013 [4]. The aim of our research was, on the one hand, to fill this gap, and on the other, to obtain information from a specific population segment: mothers of children aged 10 years or below. In Eastern European societies, where nuclear two-parent-families are predominant, mothers are the main caregivers of children [5]. In addition, full-time female employment is typical in the region, and attitude surveys carried out among the youngest employees reveal a strong determination among future parents for full-time employment [6]. That is, policies have to consider a dual earner (and, increasingly, a single parent and single earner) family type, with mothers as the main caregivers and mediators of cultural capital within the family. 

Caregiver health literacy directly impacts the health of children, as underscored by international research results [2]. The present paper is an attempt at situating the health literacy of ethnic Hungarian mothers in Eastern Europe within an international context. Health literacy is discussed mostly with regard to its antecedents and consequences. In this paper, we address the topic of antecedents and search for the socioeconomic and demographic determinants of health literacy in the region.

Unlike the medical view of health literacy, the population view and one of its best-known theoretical approaches distinguishes between three aspects of health literacy: the functional, the interactive and the critical dimensions [7]. Similarly, the theoretical base of the concept as understood in this research belongs to this population view and was formulated by the European Health Literacy Project. This integrated model of health literacy encompasses access, understanding, appraisal and application of health-related information in three domains: health promotion, disease prevention and health care [8]. Based on this departing model, the European Health Literacy Project undertook empirical research on the continent, which made up for a considerable deficiency in the field. 

Overall, there is a substantial social gradient in the health literacy of Europeans: financially deprived, have-not groups, and people with low education and old age are overrepresented in the category with limited health literacy [4]. Furthermore, the comparative analysis across countries revealed considerable regional differences, and the only Eastern European participant, Bulgaria obtained lower-than-average results. Measured with the same tool (the HLS-EU-47) at later dates, other countries in Eastern Europe also performed under the average. The mean score in Poland was lower than that of Western European countries [9], and Czech Republic occupied the penultimate place in the rank of countries involved [10]. Based on a common history of some centuries, and particularly of the past decades, one can infer that in the ethnic Hungarian population, too, the proportion of people with limited health literacy is higher than in the Western parts of Europe. The primary aim of the study is to assess the level of health literacy of ethnic Hungarian mothers in three Eastern European countries (Hungary, Romania, Slovakia) with two widely used measures and to identify the socioeconomic and demographic determinants of health literacy. 

The ethnic Hungarian population in the Carpathian Basin of Eastern Europe identifies itself as a culturally, linguistically and politically homogeneous population sharing the idea of national unity despite having lived in different countries since the border modifications connected to the peace treaties following World War I. Hungarians in Romania and Slovakia, the largest minority groups in these countries, share the idea of a Pan-Hungarian ethnocultural nation and define themselves as a part of it [11]. Further communities of ethnic Hungarians live in Serbia and the Ukraine; however, these are much smaller in number. An additional concern of considering these two latter communities for the present research is that ethnic data with respect to mortality and life expectancy are unavailable for these areas. By contrast, in Romania and Slovakia, mortality rates and life expectancy of the Hungarian minority have been studied and are acknowledged to be similar to the majority, with no significant ethnic disparities [12,13]. Unlike Serbia and the Ukraine, the three countries in which our study took place, Hungary, Romania and Slovakia, are all members of the European Union, with relatively similar living and social conditions, and belong to the so-called post-socialist welfare system type [14]. 

The above considerations explain the rationale for the sampling method. Extending the research to the ethnic majority Romanians and Slovaks is currently impeded by the non-existence of validated health literacy measures in these languages, a shortfall of health sciences in the region that will hopefully be made up for in the near future. 

### Variable Selection: Socioeconomic and Demographic Determinants of Health Literacy

Next, we will present, in short, the socioeconomic and demographic antecedents that presumably determine the level of health literacy in ethnic Hungarian mothers in the Eastern European region.

Although the methodology among studies is different, there are some unequivocal results that indicate that parents’ health literacy is also largely dependent on their educational level and socioeconomic status [2,15]. Measured with the same instruments as in the present research, educational attainment and net household income proved to be important antecedents of health literacy in Europe and beyond the continent [16,17,18,19,20,21,22,23]. Interestingly enough, in the latest research on the Polish population in Eastern Europe, there was no direct association between socioeconomic status and health literacy. However, vocational status, which had proven significant, was an indicator of socioeconomic situation [9]. 

The place of residence has only been found to be significant in Ghana [21], in the sense that the bigger the settlement, the better the health literacy scores. Such an association was, to our best knowledge, not found in other parts of the world. 

In general populations and specific patients’ samples on all continents, most studies found a negative association between age and health literacy [9,18,20,22]. However, there is some indication that health literacy might improve with age [17,23]. Results are contradictory when it comes to the relationship between the age of mothers and their health literacy: some assessed that older mothers have better knowledge on child health issues [24], while others found no age effect [25].

In some countries, such as Egypt and the Netherlands, the most important predictor of health literacy was gender, with men having significantly lower scores than women [19,23]. The same difference does not stand, however, for most European countries [9,16]. In an elderly East-German population, women had significantly lower health literacy scores than men [17]. As our research was carried out among mothers only, the main caregivers of children in our society, measuring gender differences in this population was not our intention.

In the same manner, research reports on the disadvantages of ethnic minority groups in health literacy [26,27,28]. Due to the sampling design of our research, the impact of ethnic minority membership was not the focus of attention, as there was no base for comparison with the ethnic majority populations (Romanians, Slovaks). 

With respect to parent health literacy, besides the above determinants suggested by international studies, there are some further socioeconomic and demographic antecedents worthy of consideration. Among these is the idea that a single parent has a detrimental effect upon parent health literacy [2,28]. Marital status was a significant predictor in Poland and in Ghana [9,21]. In our analysis, we approached partnership status in the simplest way possible, namely whether the respondent was a single parent or she raised her child(ren) with her husband/partner. 

In some studies, where the issue was addressed, the number of children showed negative correlation with maternal health literacy. In particular, household overcrowding was found to be significant in this respect [29].

The aim of this paper is to assess the determinants of maternal health literacy in the target population, and to the extent it is made possible by the research designs, to place the health literacy of ethnic Hungarian mothers in an international context. However, due to the differences in methodologies, only careful comparisons with results from other countries are included. 

## 2. Materials and Methods

### 2.1. Research Design

Our study addresses the issue of health literacy and its inequalities in ethnic Hungarian mothers in Hungary, Romania and Slovakia. Our cross-sectional survey was implemented at the end of 2019. The anonymous questionnaire was made available online in all three countries with the permission of the group moderators, and members of mother groupings on social media sites were encouraged to respond. The only limitation was that they were the caregiver of at least one child aged 10 years or younger. 

### 2.2. Data Collection

At the beginning of the questionnaire, our adult respondents were informed about the data collection purpose and about the fact that their answers would be processed for statistical purposes. After having read this information, interviewees gave their consent to filling in the questionnaire and then proceeded to completion. In order to minimize the selection bias inevitably resulting from the opt-in survey design [30] and thus to increase the generalizability of our results, we used iterative proportional fitting by weighting the data according to age, education and geographic region.

### 2.3. Instruments

This study is part of a larger research project carried out on various topics among ethnic Hungarian mothers. The questionnaire included standardized items related to general health literacy and to the factual knowledge component on several health dimensions, on the information sources of mothers with respect to child health issues, and on the infringement of patient rights. Finally, it inquired about the basic demographic and socioeconomic features of respondents. 

For the purpose of this paper, the results obtained with two widely used health literacy measures in general populations were analysed. The Brief Health Literacy Screen (BHLS) is a short measure for rapid screening and consists of three questions to obtain information on the following: confidence in completing health forms, the frequency of requiring assistance with reading health materials, and experiencing problems learning about a medical condition because of difficulty reading health materials [31].

The second measurement tool was the standardized questionnaire of the Health Literacy Europe research group. The survey was developed on the continent in the 2010s, originally in a longer version named HLS-EU-47 [4]. Later, however, due to its extensive length, a shortened version of the scale comprising only 16 items, the HLS-EU-16, was adapted and validated. 

The rationale for having chosen these two measures of health literacy are threefold: theory-driven, methodological and practical. 

First, the HLS-EU conceptual model, in particular, is an integrative approach with an up-to-date theoretical framework. From the very beginning, its questionnaire was elaborated for general populations, and it unites the benefits of other widely used and acknowledged measures of health literacy, offering a complex multidimensional approach [8]. Next to this, the BHLS is the most suitable rapid test for screening for inadequate health literacy [31], and as such, analysis based on this tool can substantially contribute to the identification of the population segments most in need of skills development. 

Second, the Hungarian versions of the two questionnaires have been validated in general populations and are suitable for a population-based study, even under the conditions of an online research design with a self-administered questionnaire. Two further options could have been considered. The S-TOFHLA test, measuring functional health literacy, was also validated in Hungarian language in the general population [32]; however, due to its excessive length it was considered inappropriate within the present research context. In addition, the Newest Vital Sign questionnaire, used in a previous study in a Hungarian city, reported high levels of non-responsiveness, even though interviewees were assisted in its completion [33]. 

Third, one reason for the questionnaires’ choice is also quite practical. Both questionnaire blocks are widely used across Europe and beyond and they are relatively short to maintain interviewee attention and to allow further issues to be addressed. These questionnaires are popular and frequently used in different population segments, particularly in a longitudinal study in a Hungarian city and its segregates with socially disadvantaged populations [34]; thus, our data will allow for comparisons and farther reaching inferences with policy implications in the future. 

When calculating the scale scores, a standard procedure was followed. In the case of the BHLS, after reverse-scoring the item addressing confidence with forms, responses to the three items were summed, so that the final scores ranged between 3 and 15, with higher scores indicating higher subjective health literacy. The range is divided into lower (3–9) and higher (10–15) categories, indicating inadequate and adequate health literacy, respectively, following standard procedure [32,34,35,36].

The BHLS was initially developed for ease of administration in clinical settings. Studies support its internal consistency, predictive ability and concurrent validity [34,36,37]. The measure is also applicable in general populations, as one of the best rapid screening tools. It was validated on general populations in several settings [36]. In Hungary, the measure was first used and validated on a convenience sample and on a stratified, nearly representative sample of the general adult population [32]. In our research, a reliability test was performed on the questionnaire items, and Cronbach’s alpha indicated that the scale had acceptable internal consistency (α = 0.704).

The HLS-EU-16 questionnaire was validated in several European countries: in the Catalan population [18], Italy [38], Spain [39], Iceland [40], France [41], Belgium [42], as well as on a sample of the low-health-literate Dutch population [43]. It was also validated in European immigrant populations, in an Arabic population in Sweden [44] and in Somali women in Norway [45]. In Eastern Europe, the scale was validated on a representative sample of the general parent population in Hungary [46] and in the adult population of Poland [9]. Even outside Europe, mostly in Asian countries, this short measure was validated and used in general populations in Israel [47], Indonesia [48] and Turkey [49] as well as in an outpatient setting in Egypt [19]. In all cases, the scale provided proof of good psychometric properties.

In calculating the HLS-EU-16 scores, we proceeded following the recommendations of the HLS-EU research group and according to the methodology of some previous studies [9,38,47,50]. Original 5-point Likert-type answers were dichotomized into two scores, “easy” (“fairly” or “very” easy = 1) and “difficult” (“fairly” or “very” difficult = 0). The scale score was calculated as the non-weighted sum of the subscores on each item, with a final range between 0 and 16 and higher scores indicating better health literacy. Missing values were estimated with a maximum likelihood algorithm for persons who had at least 14 valid answers, while respondents with more than two missing answers were excluded from the analysis. The internal consistency of the items as measured by Cronbach’s alpha was adequate, the scale was reliable (α = 0.763), and all items were worthy of retention. From the final scores, three levels of health literacy were defined: inadequate (0–8 points), problematic (9–12 points) and adequate (13–16 points). 

For the HLS-EU-16 scale items, where it was possible to choose “I don’t know” as answer, missing values were estimated with maximum likelihood algorithm for persons who had such answers for less than 20% of items, that is, to 3 questions. Respondents with “I don’t know” answers to more than 3 items were excluded following the standard procedure [4]. 

For both scales, a reliability test was performed on the questionnaire items. Cronbach’s alpha values indicate that the BHLS-scale and the HLS-EU-16 scale have acceptable internal consistency (α = 0.704 and α = 0.763, respectively). Exploratory factor analyses were performed with polychoric correlation that assumes categorical data structure. The BHLS items loaded high on a single factor, whereas for the HLS-EU-16 items two factors were extracted. However, most items loaded relatively high on the factor solution that consisted of all 16 items. Subsequently, scale validity was checked with confirmatory factor analysis, and the one-factor model fit was found acceptable for both measures. The comparative fit index in R was higher than 0.95, RMCA lower than 0.05, and SRMR lower than 0.08. As a result, the one-factor solution for both scales proved to be appropriate to measure the health literacy of respondents.The two measures of health literacy also correlate with each other significantly, which provides further evidence for the good psychometric properties of the scales.

### 2.4. Statistical Analysis

Descriptive characteristics reveal the main first-glance results, the answer distributions and the levels of health literacy. Thereafter, two linear regression models were fitted, one for each scale (the BHLS and the HLS-EU-16). In order to exclude possible biases due to the arbitrary nature of the cut-off points assigned to the scales, we considered it best to include the two scales in their original continuous forms in linear regression analyses. These multivariate models measured the explanatory power of socioeconomic and demographic determinants on health literacy controlled for covariates. Alleged antecedents were selected based on the review of the literature and on the results of previous empirical studies, as presented in the section on variable selection. It must be noted that the variables regarded as potential predictors should be relatively independent of each other, as mutual correlations would decrease the explanatory power of the models. Thus, socioeconomic status was approached with the subjective SES, ranging from 1 to 10, its appropriate measure in linear analysis, without further considering educational attainment, occupational status or net income.

The explanatory variables introduced in the regression models and their measurement levels were age (continuous), partnership status (categorical), socioeconomic status (ordinal), number of children (continuous), and size of the place of residence (ordinal). Age is a priori important in health research and thus remains in the regression model throughout the whole model-building algorithm. Size effects (f^2^) were calculated for the entire models and for those variables that produced significant effects.

The ordinal variable with less than 10 categories, the size of the place of residence, was examined in order to check whether to use it as a continuous variable or as a series of categorical dummy variables. The first one assumes a linear effect, which might be a much too rigorous restriction, while the latter makes use of the hierarchy between categories. Technically, the decision was made as follows: in the final regression models for both health literacy scales, two models were fitted; in the first case the size of the place of residence was used as continuous, and in the second one, as categorical variables using dummies. The more parsimonious model was chosen using the AIC information criteria indicator, so that in the final models for both scales, the variable was utilized in its original ordinal level form.

When performing the linear regression, we aimed to find the best fitting and the most parsimonious causal model, using the variable selection procedure. The model was first simplified leaving out the non-significant interaction effects one by one, following suggested methodology [51]. Then, an automated variable selection algorithm was used. Apart from age, variables were allowed to be sorted by the algorithm. The enter selection method was used, with the threshold set at 5% for inclusion and at 10% for exclusion. We subjected the final model to multicollinearity diagnostics, monitoring the VIF and tolerance indicators. 

## 3. Results

### 3.1. Sample Characteristics 

1106 mothers provided fully completed questionnaires. The answers of those respondents who perform a health-related profession were excluded from this analysis, so the sample size was reduced to 894. Our analysis only entailed women, the main caregivers for children in Eastern European societies. The age of respondents ranged from 20 to 47 years, with a mean of 35.6 years. The average family size was 3.9 persons, and the mean number of children was 1.8, similar to national trends. A total of 95.6% of mothers were married or partnered, and only 4.4% were single mothers. 

Distribution across place of residence was as follows: 22.4% urban with more than 100,000 inhabitants, 22.1% urban from 20,000 to 100,000 inhabitants, 15.3% urban with less than 20,000 inhabitants and 40.2% rural. 

Subjective social status measured on a scale of 0 to 10 resulted in a mean of 7.08. Most respondents had a university degree (71.8%), and only a minority stopped after high school or primary school (26.7% and 1.5%, respectively). As far as occupational status was concerned, 59.6% of respondents were employed, 35.1% were on maternity leave, 1.5% were still enrolled in education, 0.7% were unemployed and 5.1% were housewives. These data suggest that our sample was somewhat more affluent, with higher educational attainment and better labour market integration than the general population. This bias is due to the survey design and the online data collection procedure. In order to minimize it and to render our results more generalizable, iterative proportional fitting was used; that is, the sample was weighted on age, education and geographic region. 

A total of 14% of mothers were caring for at least one child suffering with some kind of chronic disease that required regular medical visits, which renders addressing the issue of parental health literacy even more important.

Table 1 displays the basic socio-demographic characteristics of the sample across the three countries. The only somewhat more remarkable difference is with respect to employment status, since in Hungary and Slovakia maternity leave may last for up to three years, whereas in Romania it lasts only up to two years. Thus, in our target population of mothers with children aged under 10 years, there are more mothers from Hungary and Slovakia on maternity leave and less employees. On the whole, our subsamples are very similar to each other and allow for an analysis that treats this population segment as a unitary group.

### 3.2. Descriptive Statistics

As first measures of health literacy, the distribution of answers obtained in each individual item of the two scales is displayed. To start with, Table 2 shows the distribution of answers to the BHLS items. 

The items of the second measure, the HLS-EU-16, resulted in the answer distributions as specified in Table 3.

After computing the BHLS scale from the items, a measure with a theoretical minimum of 3 and a maximum of 15 points was constructed, where respondents reached a mean of 11.62 (SD = 2.03). On the HLS-EU-16 scale ranging from 0 to 16, the mean was 12.1 (SD = 2.99). 

Following categorization of the scale scores as suggested by the literature, the two measures reveal the levels of health literacy in ethnic Hungarian mothers. Respondents were grouped into categories following standard procedure: grouping the BHLS scale into two groups [32,34,35,36] and grouping the HLS-EU-16 scale into three categories [9,38,47,50].

**Figure 1:**
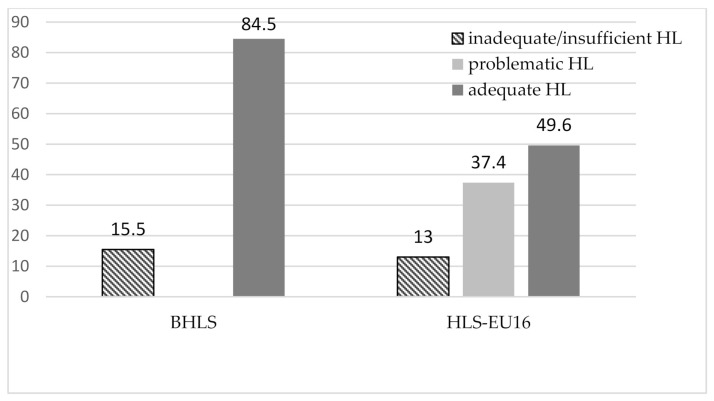
Health literacy levels of ethnic Hungarian mothers in Eastern Europe according to the two measures, *N* = 894.

As seen in Figure 1, the rate of inadequate health literacy is similar in both scales. These descriptive results, however, do not allow for far-reaching conclusions. Our aim is to identify the socioeconomic and demographic determinants of health literacy, controlled for other effects.

### 3.3. Assessing the Socioeconomic and Demographic Antecedents of Health Literacy in Mothers 

Two linear regression models were called upon to measure the impact of alleged explanatory variables one by one, adjusted to covariates. Both health literacy scales were used in their original continuous form. For both, higher numbers indicated better health literacy. 

Socioeconomic and demographic variables introduced in the regression models explain a total of 12.3% and 7.8%, respectively, of the variance of health literacy. Cohen’s f^2^ values reflect a medium explanatory power (effect size f^2^ = 0.14) of the regression model explaining the BHLS scores and a rather small explanatory power (f^2^ = 0.08) of the HLS-EU-16 regression model. In both cases, the F statistic is significant, and neither VIF nor tolerance values indicate multicollinearity among variables. Table 4 displays the models with the largest explanatory power and the variables with significant effects for both scales.

The two models provide partly consequential, partly inconclusive evidence with regard to the antecedents of health literacy. Subjective socioeconomic status (SES) is present in both models among those few socioeconomic and demographic variables that were identified as significant determinants. Measured with the BHLS, however, educational attainment has only the second biggest effect (β = 0.092) and a smaller effect size (f^2^ = 0.02), as partnership status precedes it (β = 0.103, f^2^ = 0.03). Single mothers score, on average, one point less (B = 1.051) on the scale than do married/partnered ones. Socioeconomic status also produces disparities in health literacy (B = 0.086), so that with its increase health literacy scores also improve. 

Using the HLS-EU-16 scale, the effect of socioeconomic status is the largest (β = 0.210, f^2^ = 0.43), followed at a distance by the significant impact of age (β = 0.095, f^2^ = 0.06). This model produces higher effect sizes; for socioeconomic status, there is an impressively high effect size compared to the predictors of health literacy as measured with the brief health literacy survey (BHLS). 

Apart from these variables, two for each model, no other variable that was assumed to produce disparities in parental health literacy was proved to have a significant impact when adjusted to the covariates.

## 4. Discussion

Although results obtained in several countries or settings are available, in most cases, a methodologically fair comparison is not possible due to the differences in the research designs or settings or sometimes even due to the differences in the coding of items. It is once again worth noting that the generalizability of our results is limited due to the opt-in survey design. Respondents completed the questionnaire online, so we most probably reached mothers with relatively better socioeconomic conditions than the average Hungarian parent population. However, this deficit was to a large extent emended thanks to the weighting of the data.

Keeping in mind the above considerations, one can state that in the international studies that proceeded with a similar methodology, the health literacy scores were about the same or somewhat better. On the BHLS, the mean score of health literacy in Hungarian parents was 11.62 (SD = 2.03). In inpatient and outpatient settings in the United States, haemodialysis patients reached a mean score of 11.6 (SD = 3.0) [36], whereas the mean ranged between 12.1 and 13.9 when administered to another sample of hospital inpatients and a clinic sample [34]. Thus, the health literacy of ethnic Hungarian mothers in Eastern Europe seems to be, on average, a little worse than in patient populations in more developed countries.

The HLS-EU-16 measured a mean of 12.1 (SD = 2.99) in our sample. With the same tool, a mean of 12.99 (SD = 3.11) was measured in the Polish population [9], which is similarly an Eastern European one. That result is almost one point higher than in our sample. This might suggest that Hungarian mothers are less confident about their health competence than the Polish population on the whole. This is so in spite of similar health indicators in the region, the common history of the countries, and similar social, political and cultural challenges in the recent past. 

Besides the mean scores, the population with inadequate health literacy is possibly even more important to consider. Again, although international comparisons are not totally justified, we would like to provide an estimate of the position where these measures place the health literacy of Hungarian mothers in a global perspective. 

In total, 15.5% of Hungarian mothers had inadequate health literacy as measured with the BHLS. In Europe, in general populations, 12.3% was measured for Italy and 18% for Switzerland. In Asia, Lebanon had 19.2% of the population with inadequate health literacy, whereas in Turkey, the rate reached as high as 63% [52]. In the United States, 14.5% displayed inadequate health literacy [35], and in a sample of United States haemodialysis patients, 22.8% were found to belong to this category [36]. In Australia, among ischaemic heart disease patients in an outpatient setting, 14.3% inadequate health literacy was measured [22].

In ethnic Hungarian mothers, the rate of inadequate health literacy is 13% when measured with the HLS-EU-16 scale. In the very first European study carried out with this methodology [4], 12% of the population displayed insufficient health literacy for the whole of the eight participating countries. There are limited comparison possibilities with results of European studies that were carried out with similar methodology and score calculation. In Germany, the inadequate health literacy in the general population was 12.3% [16], in Catalonia 10.3% [18], and in Italy, more precisely in Florence and the surrounding area, 11.8% [53]. 

From these careful comparisons, one can infer that ethnic Hungarian mothers in Eastern Europe display a somewhat lower health literacy than the population of more developed countries. Adding to this the bias resulting from the research design, namely that mothers in our sample are slightly more affluent than the countries’ average, the results allow us to conclude a health literacy lag of Hungarian mothers. 

The main aim of this study was to assess socioeconomic and demographic determinants of parental health literacy and the extent of their impact. This was achieved through the linear regression models fitted for both measures. 

Socioeconomic status had a significant effect, so that the health literacy scores of more affluent (and certainly, more educated) mothers were higher than those of their counterparts from a less advantageous social background. Considering two mothers on the extreme poles of socioeconomic status, their difference in health literacy was considerable ((15 − 3) × (0.086) = 1.03 points) on the BHLS and immense ((16 − 0) × (0.317) = 5.07 points) on the HLS-EU-16. Indeed, socioeconomic status measured in one way or another was the only universally and unequivocally significant variable, and in most cases, the strongest predictor of health literacy in general populations [16,17,18,19,20,21,22,23] and in parents [2,15]. 

Between mothers with similar socioeconomic background, age in itself can make a difference. Linear regression fitted for the HLS-EU-16 scale reinforced the impact of age on health literacy. Most studies from all continents found that health literacy decreased with age [9,18,20,22]. In Europe, only a few results in general populations from the Netherlands and the former Eastern Germany predicted health knowledge in line with our findings, in that it increased with age [17,23]. The reason for the positive correlation between age and maternal health literacy was that older mothers tended to overconsume health information [24], even though in the recent past, age was found to be non-significant for some dimensions of maternal health literacy [25]. Our results showed for one of the measures that the older a mother was, the more confident she was in health-related issues, and this was so even independently of socioeconomic status. 

Family structure (partnership status) was not often thematised as a health literacy determinant. However, some of the studies that addressed this point found marital status to be a significant antecedent of low health literacy in Poland [9] and in Ghana [21]. Specifically, being a single parent was negatively associated with parental health literacy in some settings [2,28]. Our results were in line with these findings, partnership status of mothers was found to be associated with health literacy of mothers. According to the results of the linear regression model for the BHLS, single mothers’ health literacy was significantly lower than that of married/partnered mothers who shared caregiver responsibilities within the family. The HLS-EU-16 measure was not sensitive to the family structure.

Some of the international research found the number of children to negatively correlate with parental health literacy [29]. Although we expected a similar result, in our models, this variable was not significant, most probably due to its correlation with socioeconomic status, which itself is responsible for many of the disparities. Similarly, the association of health literacy and socioeconomic status might have diminished the effect expected from the size of the place of residence.

Our results have policy implications for the region. Particularly in Romania and Slovakia, where, to date, health literacy has not been addressed, this research can be considered a pilot study on the topic. Even if the study only encompasses mothers of Hungarian ethnicity, and its generalizability is limited, results point at the strong social determination of caregiver health literacy. In these post-socialist countries, where socialization is predominantly taking place within the family, mostly through mothers, our results have high relevance, particularly by identifying social groups at risk of limited health literacy. 

Our countries face severe social problems during the transition to market economies. Compared to other European societies, Romania displays a high rate of poverty [54], and, in parallel, our results reveal low health literacy among the poor. Furthermore, addressing the limited health literacy of young mothers is also relevant, as the prevalence of teenage pregnancies is above the average in all three countries [55], and our data shed light on the correlation of maternal age and health literacy, a new and untypical finding in most settings. Moreover, our study identified single mothers as at risk of limited health literacy. From the countries involved in our study, Slovakia and Hungary in particular display high risk of income poverty of single parents [56]. In the population segments that our research identified as vulnerable, improving caregiver health literacy is crucial for child health and wellbeing.

## 5. Limitations

In this study, there are a number of limitations to consider. First, the potential for the generalization of results is somewhat limited due to the online sampling design. That is, our results are presumably skewed upwards, being better than the results we would have obtained with classical paper-based questionnaires or face-to-face interviews from a more heterogeneous group of respondents, including those that are absolutely not reachable online. This limitation was, at least partly, corrected by iterative proportional fitting in statistical analysis. A second hardship is posed by the limited explanatory power of the regression models. Although not rare in social sciences, the adjusted R^2^ values are certainly not large, and they suggest that besides the sociodemographic characteristics studied in this paper, health literacy is impacted by a range of other factors, unexplored in this study or as yet unknown. In the future, based on the data already gathered and as far as is possible, further potential determinants of health literacy in mothers can be sought, such as health conditions in their children or the sources of information they access on child health topics. Third, only ethnic Hungarian mothers were considered from three of the Eastern European countries they live in, without results from their co-citizens in the ethnic majority; this was due to a lack of validated measurement tools in those languages. This deficiency will be addressed in the near future by a group of health researchers by translating and validating health literacy measurement instruments into the Romanian and Slovak languages. 

## 6. Conclusions

This study provides some insights into the levels of health literacy in ethnic Hungarian mothers in Eastern Europe. Placed in an international context, our countries seem to score lower than other European countries and the United States but higher than other continents. In addition, in this region, health literacy is, to a large extent, socially determined. Socioeconomic and demographic factors influence the health literacy of mothers. Some allegedly important predictors, such as the number of children and the size of the place of residence, have no impact in themselves, while others lead to considerable differences. Most importantly, there are immense differences between mothers with low and high educational attainment, between younger and older mothers, and between single and partnered mothers. 

Our analysis reinforces the role of socioeconomic capital, widely recognized to be associated with health literacy in general and with parental health literacy in particular. Disparities in health literacy reflect, to a large extent, socioeconomic inequalities. We consider that the key findings of our study are the improvement of health literacy with age and revealing the hardships of single mothers. These issues should be further addressed when targeted population interventions are designed. 

## Figures and Tables

**Table 1:** Sample characteristics across the three countries, *N* = 894.

Variable	Hungary (*N* = 317)	Romania (*N* = 341)	Slovakia (*N* = 236)
Age (mean)	36.7	34.2	35.6
Average family size (mean)	3.9	3.8	3.9
Number of children (mean)	2	1.7	1.9
Marital status: single (%)	4.5	4.3	4.2
Urban, >100,000 inhabitants (%)	24.8	20.1	18.6
Urban, 20,000–100,000 inhabitants (%)	23.2	22.1	19.1
Urban <20,000 inhabitants (%)	11.9	15.5	17.9
Rural (%)	40.1	42.3	44.4
Subjective SES (0–10, mean)	7.36	6.98	7.16
Low educational attainment (%)	1.2	1.9	1.6
Middle educational attainment (%)	25.3	26.9	26.4
High educational attainment (%)	73.5	71,2	72
Employee (%)	55.9	59.1	56.2
On maternity leave (%)	36.7	32.9	36.1
In education (%)	1.7	1.4	2.0
Unemployed (%)	0.7	1.2	1
Housewife, full-time mother (%)	5.0	5.4	4.7
Has a child with chronic illness (%)	14.2	13.8	14.0

**Table 2:** Distribution of answers to the items of the BHLS scale (%), *N* = 894.

	Question	Extremely	Quite a Bit	Some-What	A Little Bit	Not at All
1	How confident are you filling out medical forms by yourself?	35.1	53.6	9.3	2.1	0
		All of the time	Most of the time	Some of the time	A little of the time	None of the time
2	How often do you have someone help you read hospital materials?	0	32.0	23.7	33.0	11.3
3	How often do you have problems learning about your medical condition because of difficulty understanding written information?	1.0	9.3	3.1	30.9	22.7

**Table 3:** Distribution of answers to the items of the HLS-EU-16 scale (%), *N* = 894.

	On a Scale from Very Easy to Very Difficult, How Easy Would You Say It Is to…?	Very Easy + Easy	Difficult + Very Difficult	Don’t Know/No Answer
1	Find information on treatment of illnesses that concern you	81.6	14.0	4.4
2	Find out where to get professional help when you are ill	66.9	33.1	0
3	Understand what your doctor tells you	78.7	20.6	0.7
4	Understand your doctor or pharmacist’s instructions on how to take a prescribed medicine	97.1	2.9	0
5	Judge when you may need to get a second opinion from another doctor	51.5	48.5	0
6	Use information the doctor gives you to make decisions about your illness	58.8	39.0	2.2
7	Follow instructions from your doctor or pharmacist	94.9	5.1	0
8	Find information on how to manage mental health problems like stress and depression	55.1	41.2	3.7
9	Understand health warnings about behaviour such as smoking, low physical activity and drinking too much	92.6	7.4	0
10	Understand why you need health screenings	88.2	10.3	1.5
11	Judge if the information on health risks in the media is reliable	42.6	54.4	2.9
12	Decide how you can protect yourself from illness based on information in the media	46.3	50.8	2.9
13	Find out about activities that are good for your mental well-being	86.7	13.3	0
14	Understand advice on health from family members or friends	45.6	22.8	5.1
15	Understand information in the media on how to become healthier	67.6	29.5	2.9
16	Judge which everyday behaviour is related to your health	56.6	42.6	0.7

**Table 4:** The determinants of mothers’ health literacy according to the two measures.

Explanatory Variables	B	*p*	β	f^2^	B	*p*	β	f^2^
	BHLS (Range 3–15)				HLS-EU-16 (Range 0–16)			
Age	0.017	0.294	0.043		0.074	0.018	0.095	0.006
Partnership status (single-partnered)	1.051	0.004	0.103	0.03	1.532	0.553	−0.056	
Number of children (increasing)	0.067	0.838	0.023		0.640	0.522	−0.065	
Subjective SES (increasing)	0.086	0.034	0.092	0.01	0.317	0.003	0.210	0.043
Size of the place of residence (increasing)	0.068	0.479	0.027		0.098	0.654	0.045	
Constant	10.073	0.000			8.155	0.018		
	Adjusted R^2^ = 0.123, f^2^ = 0.14, F = 0.281, *p* = 0.000				Adjusted R^2^ = 0.078, f^2^ = 0.08, F = 1.134, *p* = 0.000			

Linear regression, *N* = 894.

## Data Availability

Dataset is available upon request.
